# Quantitative estimation of optical properties in bilayer media within the subdiffusive regime using a tilted fiber-optic probe in diffuse reflectance spectroscopy, part 1: a theoretical framework for designing probe geometry

**DOI:** 10.1117/1.JBO.29.10.105001

**Published:** 2024-10-29

**Authors:** Philippe De Tillieux, Maxime Baillot, Pierre Marquet

**Affiliations:** a Cervo Brain Research Centre, Québec, Canada; b Université Laval, Department of Physics, Physical Engineering, and Optics, Québec, Canada; c Université Laval, Department of Psychiatry and Neurosciences, Québec, Canada; d Centre For Optics, Photonics and Lasers, Québec, Canada; e Joint International Research Unit, Université Laval, Centre for Psychiatric Neuroscience, Department of Psychiatry, Lausanne University Hospital, University of Lausanne, Prilly, Switzerland

**Keywords:** diffuse reflectance, effective phase function, subdiffusive, intrinsic optical properties

## Abstract

**Significance:**

As biological tissues are highly heterogeneous, there is a great interest in developing non-invasive optical approaches capable of characterizing them in a very localized manner. Obtaining accurate absolute values of the local optical properties from the measured reflectance requires finding a probe geometry, which allows us to solve this inverse problem robustly and reliably despite neglecting the higher-order moments of the scattering phase function.

**Aim:**

Our goal is to develop a theoretical framework for designing tilted-fiber diffuse reflectance probes that allow quantitative estimation of the optical properties corresponding to limited tissue volume (typically a few cubic millimeters).

**Approach:**

Relationships among probe geometry, sampled tissue volume, and robustness of the inverse solver to calculate optical properties from reflectance are studied using Monte Carlo simulations.

**Results:**

The analysis of the number of scattering events of the collected photons leads to the establishment of relationships among the probe geometry, the sampled tissue volume, and the validity of a subdiffusive regime for the reflectance.

**Conclusions:**

A methodology is proposed for the design of new compact probes with tilted fiber geometry that can quantitatively estimate the values of the optical coefficients in a localized manner within living biological tissues by recording diffuse reflectance spectra.

## 1 Introduction

Spatially resolved diffuse reflectance spectroscopy (srDRS) is a technique capable of non-invasively characterizing biological tissues *in vivo*.^1^^–^^3^ It simultaneously reveals information about the absorption and scattering properties of a tissue, the so-called intrinsic optical properties (IOPs). These properties offer relevant information about tissue histoarchitecture and physiological processes. The absorption spectra reveal key information about tissue biochemistry through the concentration of the chromophores (e.g., oxy- and deoxyhemoglobin, melanin, water content),^4^ whereas the scattering spectra offer an insight into the morphology of the cellular structure by revealing changes in the cell concentration and size distribution.^5^^,^^6^ Typical DRS setups include one illumination fiber and several collection fibers at different source–detector separations (SDSs). As the backscattered light depends on the SDSs and IOPs, an inverse problem that takes into account the geometry of the experimental set-up must be solved to recover the IOPs from the measured diffuse reflectance spectra. The forward problem of modeling the photon propagation inside the tissue as a function of the experimental set-up geometry and the optical coefficients is typically solved through Monte Carlo (MC) simulations. A common assumption in the numerical model is that the volume sampled by the light is homogeneous. However, biological tissues are organized into different specialized structures, which challenge this assumption of homogeneity. To increase the likeliness of the sampled volume being homogeneous, a usual approach is to attempt to reduce the volume sampled by the light inside the tissue to probe the tissue locally, i.e., to possibly probe a specific tissue structure. Such a reduction of the sampled volume can be done either by reducing the SDSs^7^ or by tilting the light injection and collection fibers with respect to the tissue surface.^8^ Both these strategies have the benefit of reducing the probed tissue volume, but they come at the expense of a more complex photon migration model. This is because the contribution of the scattering phase function, which describes the probability distribution of the scattering direction, to the backscattered light cannot be reduced to a limited number of its Legendre moments if the collected photons have undergone too few scattering events.^9^^,^^10^ By contrast, a very large SDS allows the use of the well-known diffusion approximation,^11^ which corresponds to a contribution of the phase function reducing to its first moment; a shorter SDS must include higher-order moments of the phase function. An empirical criterion based on the product of the SDS and the reduced scattering coefficient ($\rho \mu_{s}^{\prime }>0.5$) was shown to be effective in characterizing the limits of the subdiffusive regime using a second-order approximation of the phase function.^7^ This criterion was developed for an experimental setup using optical fibers placed perpendicularly to the tissue’s surface under a restricted range of IOPs. In this work, a more generalized criterion based on the physics of multiple scattering applicable to any geometry of the experimental set-up and any IOPs of the probed sample is proposed. This criterion can then be used to identify the minimum SDS ($\rho_{\min}$) required to satisfy the conditions of the second-order approximation of the phase function. For SDS shorter than those defined by this criterion, Legendre moments higher than order 2 will begin to contribute significantly to the backscattered signal collected. In addition, the effects of the maximum SDS ($\rho_{\max}$), resulting from the spatial distribution of the detection fibers, on both the robustness of the inverse problem to calculate the IOPs and the size of the tissue volume sampled by the backscattered light are explored. All in all, by studying these different relationships among the probe geometry (spatial distribution of the fibers, tilt angles), the maintenance of a subdiffusive regime for the diffuse reflectance, the size of the tissue volume sampled by the backscattered light, and the robustness of the corresponding IOP determination, we propose a methodology based on our multiple scattering criterion, which allows designing a probe geometry capable of robustly and quantitatively calculating the IOPs corresponding to a limited sampled tissue volume.

## 2 Physical Model: Monte Carlo Simulation, Scattering Phase Function, and Probe Geometry

**Fig. 1:**
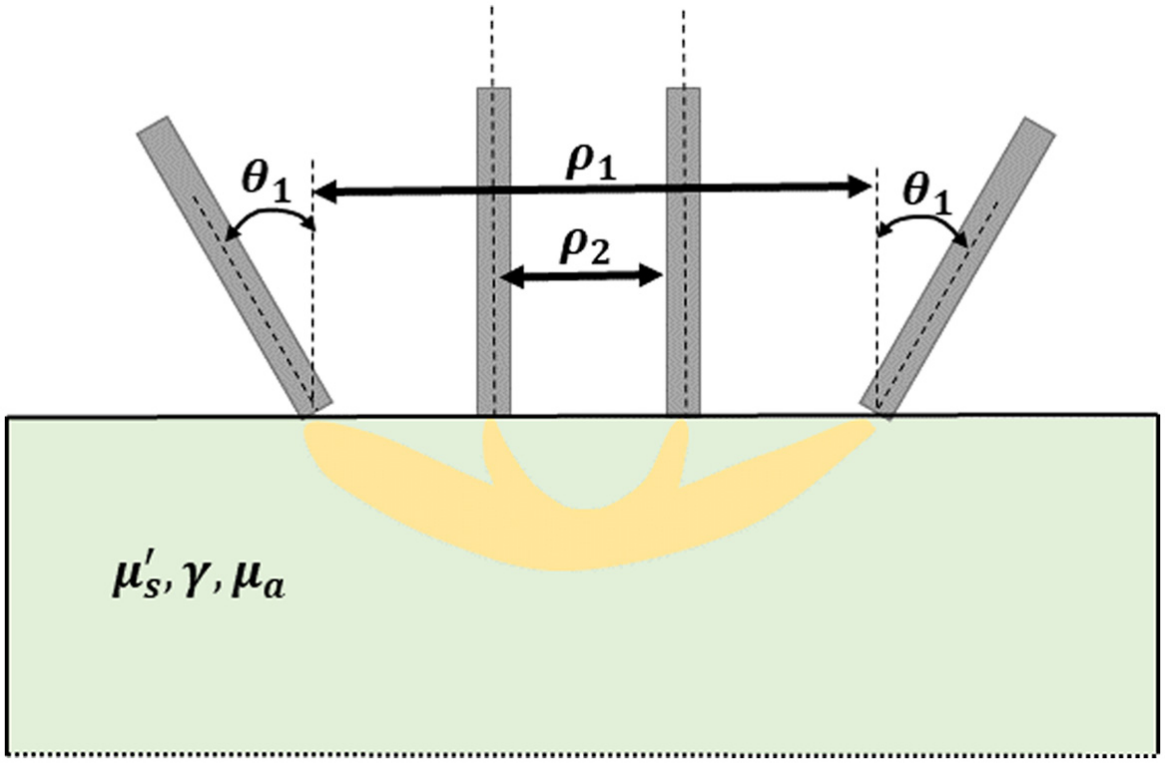
Schematic of a DRS setup using two fiber combinations. The fibers of combination 1 are at a greater SDS and tilt angle, but they result in a similar sampled depth to that of combination 2, which has fibers at shorter SDS with a tilt angle of 0 deg.

A custom Monte Carlo program^12^ is used to model light scattering inside tissues. A semi-infinite space is assumed in the simulations. A schematic representation of the geometric parameters of the problem is given in Fig. 1. This example represents two fiber combinations at different source–detector separations (ρ) and different tilt angles (θ). The tilt angle is defined with respect to the normal of the surface. Although the tilt of the illumination and detection fibers may be adjusted individually, they are here set to be equal and are tilted in opposite directions to reduce the number of parameters in the problem. The refractive index of the tissue is set to 1.43, a common assumption in the literature.^13^ The diameter of the fibers is set to 0.1 mm and the numerical aperture to 0.4, which are common values in DRS setups.^14^ In all simulations, $10^{8}$ photons are launched, which results in a mean uncertainty of ∼4% for the range of SDS considered in this work. This high number of photons is required for simulating tilted fibers because the radial symmetry that can be exploited for perpendicular fibers is broken, which implies a slower convergence of the computed reflectance.

The scattering phase function describes the probability per unit solid angle of a change of direction s^ to s^′. Under the assumption that the medium is isotropic, the scattering angle θ is independent of the initial direction, so p(s^·s^′)=p(cos θ). The moments of the phase function are defined as^15^

$$g_{n}=\int_{-1}^{1}P_{n}(\cos\;\theta )p(\cos\;\theta )d(\cos\;\theta ),$$ (1)

where $P_{n}$ is the Legendre polynomial of order n. An analytical phase function only exists for particular cases, e.g., for a dielectric sphere using Mie theory.^15^ Various phase functions have been proposed to model more complex media such as biological tissues.^10^ The Henyey–Greenstein (HG) phase function is often used^9^^,^^16^^,^^17^ because its assumption of the fractal size distribution of the scatterers is a reasonable approximation for biological tissue and because it has the further benefit of being a convenient one-parameter function for which the asymmetry parameter is given by the parameter $g_{1}=g_{\mathrm{HG}}$

$$p_{\mathrm{HG}}=\frac{1-g_{\mathrm{HG}}^{2}}{{\left(1+g_{\mathrm{HG}}^{2}-2g_{\mathrm{HG}}\;\cos\;\theta \right)}^{3/2}}\;g_{n}=g_{\mathrm{HG}}^{n}.$$ (2)

However, the HG phase function was shown to underestimate the backscattering intensity.^18^ Because the first moment determines all the subsequent moments in the HG phase function, the backscattering intensity cannot be adjusted to better represent experimental results. To correct this drawback, the modified Henyey–Greenstein (MHG) was proposed^18^

$$p_{\mathrm{MHG}}=\alpha p_{\mathrm{HG}}(\theta )+(1-\alpha )\frac{3}{4\pi }\;\cos^{2}\;\theta \;g_{1}=\alpha g_{\mathrm{HG}},\;g_{2}=\alpha g_{\mathrm{HG}}^{2}+2/5(1-\alpha ),\;g_{n}=\alpha g_{\mathrm{HG}}^{n}\text{ for }n>2,$$ (3)

where α can vary between 0 and 1. In the MHG phase function, the $g_{2}$ moment can vary independently of the first moment, but all subsequent Legendre moments are determined by the initial choice of $g_{1}$ and $g_{2}$. As we are interested in analyzing the effect of the higher-order moments ($g_{n>2}$) on the reflectance measurement, a second phase function that can mimic the first two moments of the MHG function but with different higher-order moments is used. The modified power of cosines (MPC) phase function is^15^

$$p_{\mathrm{PC}}=\frac{1}{4\pi }\frac{N+1}{2^{N}}(1+\cos\;\theta {)}^{N}\;g_{{\mathrm{PC}}_{1}}=N/(N+2),\;g_{{\mathrm{PC}}_{n}}=g_{{\mathrm{PC}}_{n-1}}(N-n+1)/(N+n+1)\text{ for }n>1\;p_{\mathrm{MPC}}=\alpha p_{\mathrm{PC}}+(1-\alpha )\frac{3}{4\pi }\;\cos^{2}\;\theta \;g_{1}=\alpha g_{{\mathrm{PC}}_{1}},\;g_{2}=\alpha g_{{\mathrm{PC}}_{2}}+\frac{2}{5}(1-\alpha ),\;g_{n}=\alpha g_{E_{n}}\text{ for }n>2.$$ (4)

The first Legendre moments give the general shape of the phase function, whereas the higher-order moments give the finer details of the phase function.^19^ As photons undergo many scattering events, the effects of the scattering phase function on reflectance can be approximated with a limited number of Legendre moments. For example, the well-known diffusion approximation corresponds to a reflectance that depends only on the first moment, which can be combined to $\mu_{s}$ through the introduction of the reduced scattering coefficient, $\mu_{s}^{\prime }=\mu_{s}(1-g_{1})$. This implies that, under the condition that photons have scattered enough times, different $\mu_{s}$ and $g_{1}$ combinations leading to the same $\mu_{s}^{\prime }$ value result in the same reflectance value. Bevilacqua et al.^14^ later proposed a way of taking into account the second moment by introducing a parameter $\gamma =(1-g_{2})/(1-g_{1})$ that depends only on the characteristics of the phase function and was shown to be related to the size distribution of the scatterers in the tissue.^20^ By taking into account a second Legendre moment, the condition on the number of scattering events required for the approximation to hold is relaxed, meaning that the approximation is valid at shorter SDS than for the first-order approximation. Higher-order approximations have also been proposed in the literature,^21^^,^^22^ but although they allow working at even shorter SDS, they have not been linked to any physical characteristic of the scatterers and they add considerable complexity to the inverse problem. With the MHG and MPC phase functions in hand, we are now in a position to study what conditions, in terms of number of scattering events, the backscattered photons must satisfy so that moments of order higher than 2 of the phase function do not significantly affect the reflectance signal. In Sec. 3.3, these conditions are defined in terms of the number of photon scattering events to form a criterion.

Using a second-order approximation, there are thus three IOPs to estimate: $\mu_{s}^{\prime }$, γ, and $\mu_{a}$. A measurement from a single fiber is not sufficient to obtain a robust and quantitative estimation of these three IOPs. Typical DRS setups measure the reflectance at different SDSs. The larger the SDS, the longer the photon path length (L) inside the tissue. A wide variety of path lengths strengthens the effect of absorption on the backscattered signal, leading to a more accurate determination of the absorption coefficient estimation. Indeed, according to the Beer–Lambert equation, the photon packets are weighted as a function of a combination of their path length and absorption coefficient: $I(L,\mu_{a})=I_{0}e^{-\mu_{a}L}$. Using fibers that collect photons with shorter and longer paths, i.e., sampling a large range of L, allows us to discriminate more clearly the effect of absorption on the signal. Increasing the SDS, however, also increases the sampled depth. A compromise must be made between the estimation robustness and the requirement for a limited probed tissue volume. This methodology for selecting $\rho_{\min}$ and $\rho_{\max}$ is intended to provide a framework for designing new DRS probes with tilted-fiber geometries that allow quantitative IOP estimation within a limited tissue volume. This allows the backscattered signal to be accurately described by the IOPs $\mu_{a}$, $\mu_{s}^{\prime }$, and γ, i.e., without a significant influence of the higher-order Legendre moments ($g_{n>2}$) and consequently to be able to robustly determine these three IOPS from the measured reflectance.

## 3 Determining the Minimum SDS: a Criterion Based on the Number of Scattering Events

### 3.1 Effect of Neglecting Higher-Order Moments

**Fig. 2:**
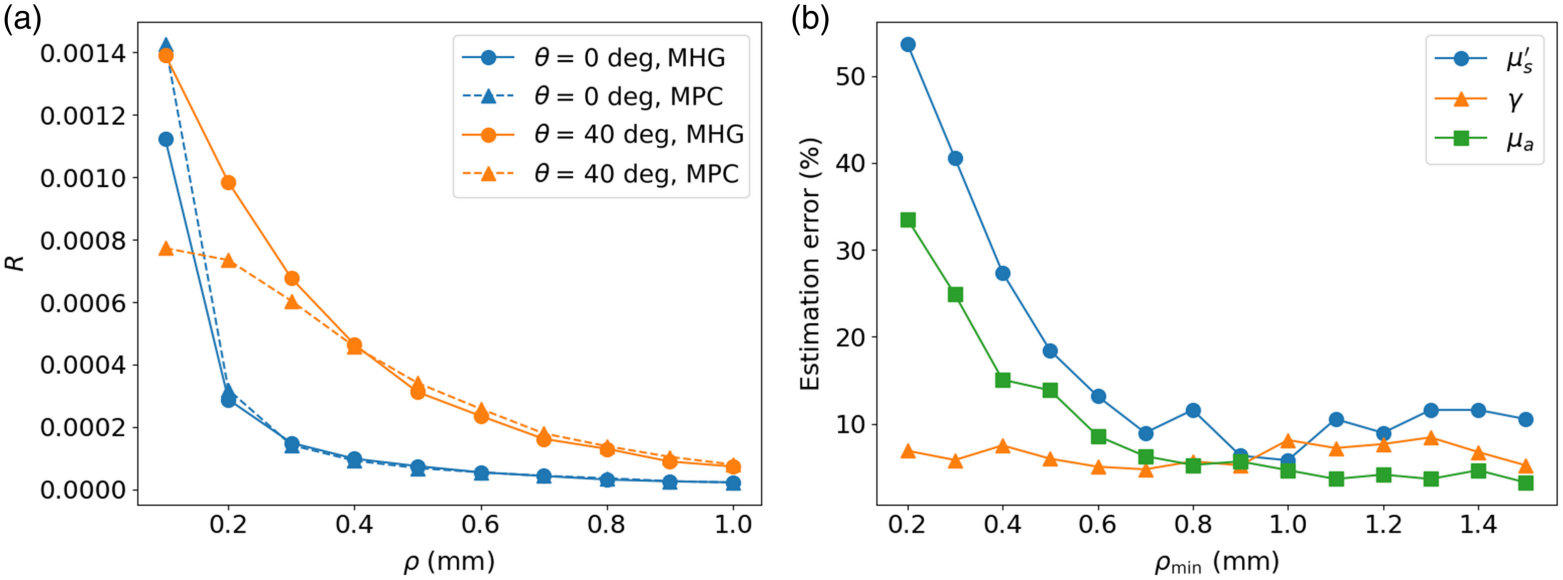
(a) Reflectance curves using the MHG and MPC phase function as a function of ρ for different fiber tilt angles. (b) Estimation error as a function of $\rho_{\min}$ with three detection fibers spaced at 0.1 mm of each other. The MPC phase function is used to generate the synthetic data, and the MHG phase function is used to estimate the IOPs.

The effect of neglecting the higher-order moments when the conditions for the second-order approximation have not been satisfied has an influence both on the forward model and the inverse solver. To show the effect of the higher-order moments on the forward problem, IOPs typical of biological tissues in the visible wavelength range are used,^13^ namely, $\mu_{s}^{\prime }=2\;{\mathrm{mm}}^{-1}$, γ=1.5, and $\mu_{a}=0.2\;{\mathrm{mm}}^{-1}$, labeled case #1. Monte Carlo simulations are performed for identical optical setups and IOPs but with different phase functions (either MPC or MHG). This guarantees that the difference in the reflectance curves is solely due to the difference in moments of order higher than 2. Figure 2(a) shows the reflectance value of each detection fiber as a function of their distance from the illumination fiber using the MHG (circle markers) and MPC (triangle markers) phase functions for fibers perpendicular to the surface (in blue) and with a 40 deg tilt angle (in orange). We see that, when the $\rho \mu_{s}^{\prime }>0.5\;\mathrm{mm}$ criterion is met (in this case, at ρ=0.25  mm), the reflectance difference remains less than 10% for fibers perpendicular to the surface, as opposed to a tilted-fiber geometry. This shows that the aforementioned criterion depends on the fiber geometry. This is because the greater the tilt, the shorter the photon path length for a given distance ρ between the illumination and collection fibers. A shorter photon path length implies that the photons have undergone fewer scattering events, so the contribution of the higher-order moments to the reflectance remains more important.

Neglecting higher-order moments for short SDS also affects the solution of the inverse problem.^23^^,^^24^ The non-linear inverse problem is usually solved by minimizing a cost function such as the relative error

$$(\mu_{s}^{\prime *},\gamma^{*},\mu_{a}^{*})=\underset{\mu_{s}^{\prime },\gamma ,\mu_{a}}{\min}\;C,$$ (5)

$$C=\sum_{\rho }\frac{\left|R_{\mathrm{sim}}(\rho ,\mu_{s}^{\prime },\gamma ,\mu_{a})-R_{\exp}(\rho )\right|}{R_{\exp}(\rho )}.$$ (6)

A cost function based on the relative error was found preferable to one based on the absolute error as the latter gives a much larger weight to the fibers at shorter SDS, which negatively impacts the absorption coefficient estimation. A quadratic function such as the root mean square error is also a common cost function choice, but it is more sensitive to measurement errors as it strongly penalizes larger errors.^23^ There are two sources of error in the computed reflectance: the numerical uncertainty from the Monte Carlo simulation and the uncertainty arising from the neglected higher-order moments. To mitigate the numerical uncertainty, a very high number of photons ($10^{8}$) was used in the simulations, which resulted in a numerical uncertainty much smaller than the one caused by the higher-order moments. It can be expected that a large error from the higher-order moments in the simulated reflectance curves impacts the solution $\left(\mu_{s}^{\prime *},\gamma^{*},\mu_{a}^{*}\right)$ of the inverse problem. To investigate the effect of the higher-order moments on the solution of the inverse solver, synthetic data ($R_{\exp}$) are generated with the MPC phase function, and the IOPs are estimated assuming the MHG phase function ($R_{\mathrm{sim}}$). To find the IOPs that minimize the cost function, a large look-up table (LUT) is generated using the MHG phase function covering a large range of realistic optical coefficients: $\mu_{s}^{\prime }\in [0.25,3]\;{\mathrm{mm}}^{-1}$, γ∈[1.0,2.0], and $\mu_{a}\in [0,1]\;{\mathrm{mm}}^{-1}$ with 40 points discretized along each axis. Synthetic data are generated for the properties of case #1 using the MPC phase function. The choice of the MHG phase function for generating the LUT and the MPC phase function to generate the synthetic data is based on the fact that the MHG phase function is generally used to construct the LUT and invert experimental data. It is more commonly used in biomedical optics as it stems from reasonable assumptions, i.e., the size of the scatterers follows a fractal distribution, whereas the MPC phase function is purely a mathematical tool to obtain identical $g_{1}$ and $g_{2}$ moments with different higher-order moments.

To visualize the effect of the higher-order moments on the IOP estimation, three detection fibers placed at $\rho_{\min}$, $\rho_{\min}+0.1\;\mathrm{mm}$, and $\rho_{\min}+0.2\;\mathrm{mm}$ are used to assess the properties of the medium. The estimation error using these three detection fibers is plotted as a function of $\rho_{\min}$ in Fig. 2(b). We see that the estimation error decreases as the minimum SDS increases. This is because the effect of the higher-order moments decreases at higher SDS, which allows for a more accurate estimation of the IOPs.

### 3.2 Legendre Moments in Multiple Scattering

**Fig. 3:**
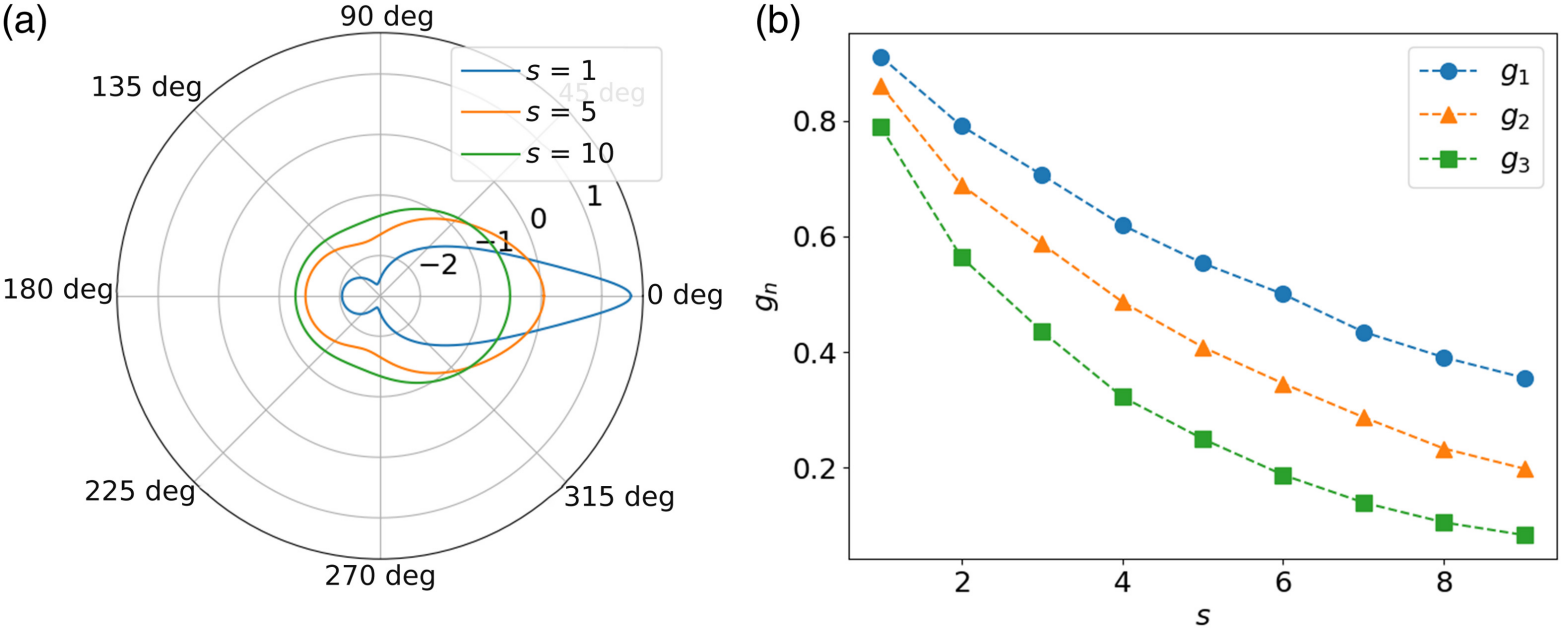
(a) Effective phase function after 1, 5, and 10 scattering events, and (b) first three Legendre moments of the effective phase function as a function of the number of scattering events.

The scattering phase function describes the angular distribution of the scattered photons in the case of a single scattering event. After multiple scattering events, the concept of effective phase function can be used to describe the resulting angular distribution of the scattered photons. The effective phase function can be calculated numerically through Monte Carlo simulations, where the cosine of the direction after two scattering events is given by^25^

$$\cos\;\theta_{n}=\cos\;\theta_{1}\;\cos\;\theta_{2}+\sin\;\theta_{1}\;\sin\;\theta_{2}\;\cos\;\phi_{1,2},$$ (7)

where $\theta_{n}$ is the resulting direction, $\theta_{1}$ and $\theta_{2}$ are the directions of the polar angles in the scattering plane of each event, and $\phi_{1,2}$ is the azimuthal angle of the second scattering event relative to the first one.^25^ To calculate more than two collisions, the process can be repeated iteratively by updating $\cos\;\theta_{n}\to \cos\;\theta_{1}$ and by sampling a new $\theta_{2}$ and $\phi_{2}$. Figure 3 shows an example of the effective phase function after 1, 5, and 10 scattering events with the first two moments of the initial phase function set to 0.9 and 0.85. It appears clear that the effective phase function tends to isotropy as light undergoes scattering events.

Another way to understand the convergence of the effective phase function toward isotropy is to observe the decay of the Legendre moments. The Legendre moments for the effective phase function can be calculated using Eq. (1). Figure 3 shows the convergence of the first three Legendre moments of the effective phase function as a function of the number of scattering events. The decay of the first Legendre moment (the mean cosine) in the multiple scattering regime has been shown to decrease as a function of $g^{s}$ where s is the number of scattering events.^26^^,^^27^ This decay of the first Legendre moment is independent of the choice of the phase function. The theoretical reason for this decay can be understood by analyzing the rotation matrix applied to model photon scattering. Through a statistical analysis of the rotation matrices applied at every scattering event, it was shown that^26^ ${\left\langle \cos\;\theta \right\rangle }_{s}={\left\langle \cos\;\theta \right\rangle }^{s}$. In other words, the mean cosine after s scattering events is equal to the mean cosine of the original phase function to the power s. The decay of the second moment can be similarly obtained^26^ from

$${\left\langle \cos^{2}\;\theta \right\rangle }_{s}=1+2{\left[\frac{3\langle \cos^{2}\;\theta \rangle -1}{2}\right]}^{s}.$$ (8)

### 3.3 Proposed Criterion

**Fig. 4:**
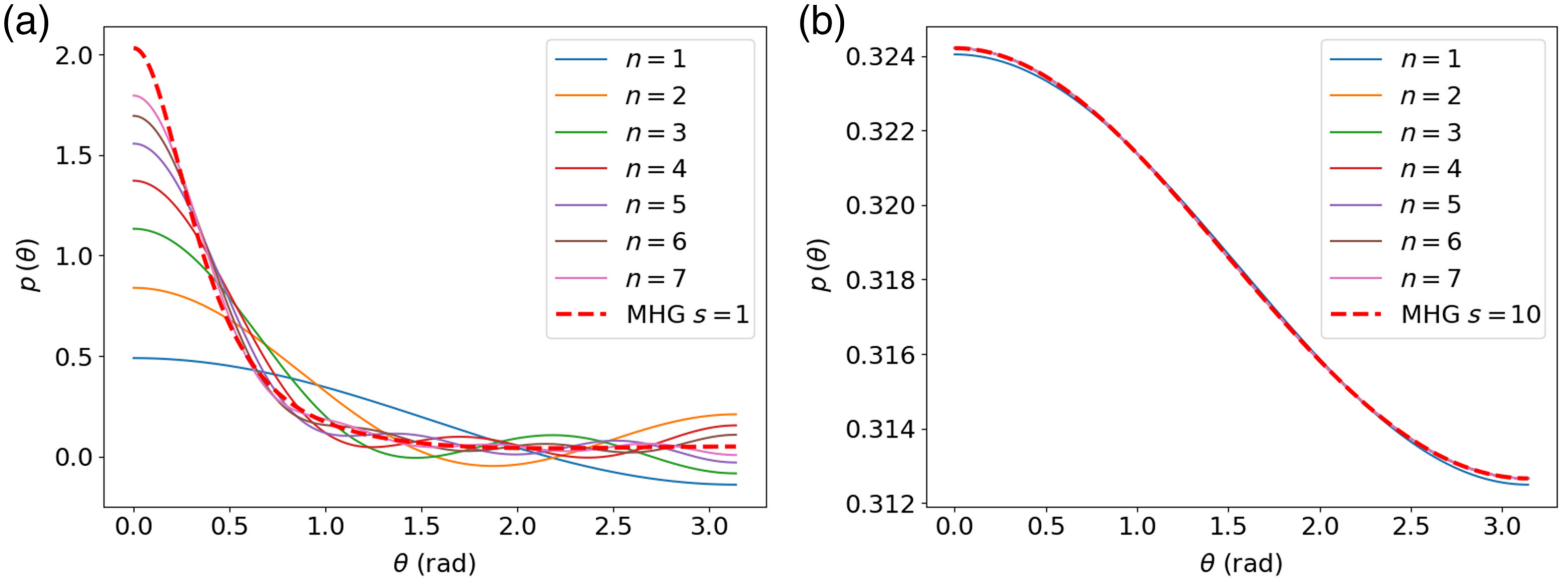
Effective phase function and approximation using a limited number n of Legendre moments after 1 scattering event (a) and 10 scattering events (b).

From the previous observations, it appears clearly that a criterion guaranteeing a minimal effect of the higher-order Legendre moments should depend on the number of scattering events of the photons collected at a detection fiber. Figure 4 compares the effective phase after one and ten scattering events expressed by the Legendre series expansion with a limited number of moments. We see that, after a single scattering event, the phase function approximation with the first seven Legendre moments remains a poor representation of the actual MHG phase function. On the other hand, after 10 scattering events, the effective phase function can be accurately represented by the first two Legendre moments.

**Fig. 5:**
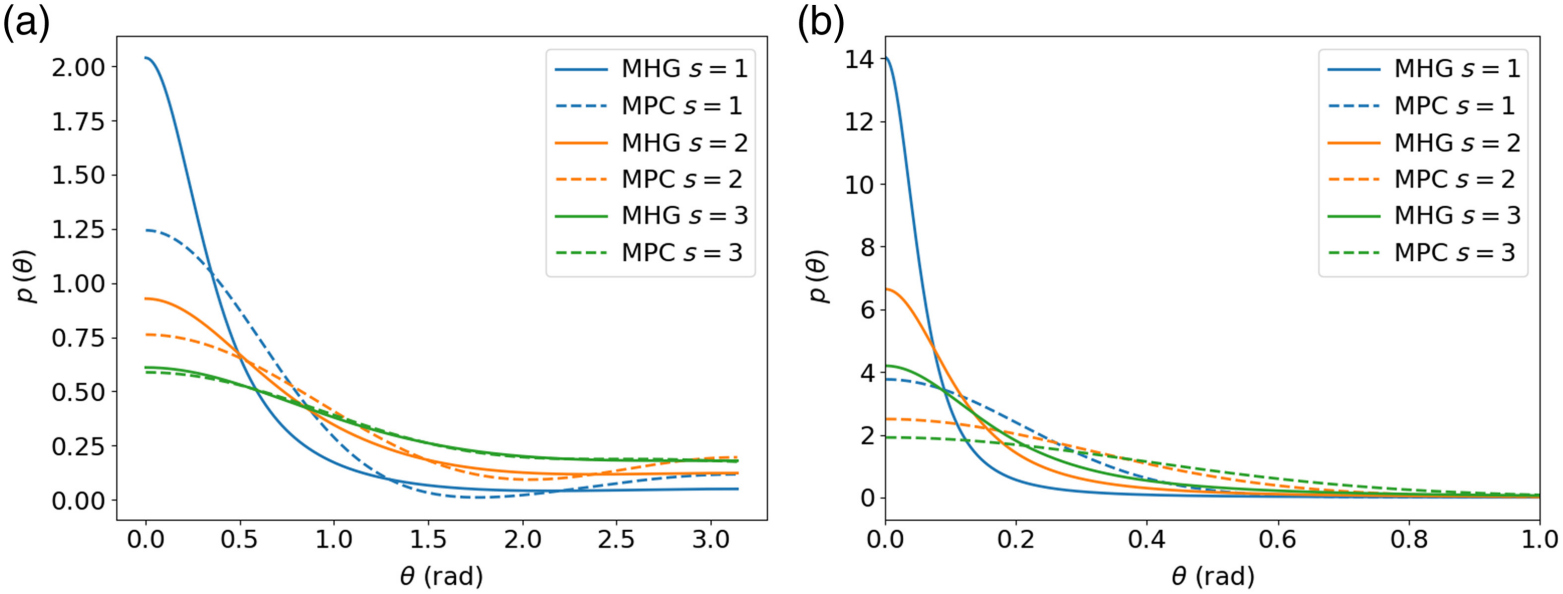
Effective MHG and MPC phase functions after 1, 2, and 3 scattering events with lower $g_{1}=0.6$, $g_{2}=0.4$ values (a) and higher values $g_{1}=0.9$, $g_{2}=0.85$ (b).

However, the convergence speed of the effective phase function toward isotropy depends not only on the number of scattering events but also on the initial shape of the phase function. Figure 5 shows the MHG and MPC effective phase functions with identical $g_{1}$ and $g_{2}$ after one, two, and three scattering events for two cases. The first case corresponds to low $\left(g_{1},g_{2}\right)$ values, which represent a flatter phase function, whereas the second case has higher $\left(g_{1},g_{2}\right)$ values corresponding to a forward-peaked phase function. We see that, in both cases, the functions converge toward each other as the number of scattering events increases because the effect of the higher-order moments decreases, but they do so at a different speed. This is because a very narrow phase function will converge to isotropy much more slowly than a flatter phase function. This can be observed mathematically in the exponential decay of the mean cosine and by the decay of the second moment in Eq. (8).

These results show that, as photons undergo multiple scattering, effective phase functions with identical first two moments and different higher-order moments converge toward each other, i.e., the impact of the higher-order Legendre moments on the reflectance decreases. This is true not only for the MHG and MPC phase functions but also for any phase function because the exponential decay of the effective phase function’s Legendre moments as a function of the number of scattering events does not depend on the choice of the phase functions. Furthermore, we know that the inverse problem, which aims to quantitatively compute intrinsic optical coefficients from diffuse reflectance, does not properly account for the contribution of higher-order moments. Therefore, we need to be able to verify that these moments have a minimal influence on the diffuse reflectance. In practice, the contribution of these moments to the diffuse reflectance shows (1) a decrease in the number of scattering events (s) and (2) a dependence on how forward-peaked the scattering phase function is. Thus, to find a criterion that allows us to estimate the contribution of these higher-order moments to the diffuse reflectance, we first must find a parameter that takes into account these two characteristics while being easy to use in MC simulations. For this purpose, we propose to calculate for each photon detected after s scattering events in a reflectance MC simulation, a weight $W_{s}$ defined as the sum of the differences of the higher-order moments between the two s-scattered MHG and MPC effective phase functions with identical $\left(g_{1},g_{2}\right)$ moments, corresponding to those used in the MC simulations. Due to the identical first two moments, $W_{s}$ can simply be calculated from the difference in area between the two s-scattered effective phase functions according to the following equation:

$$W_{s}=\int |\overset{\infty }{\underset{n=3}{\sum }}(g_{n,\mathrm{MHG}}^{s}-g_{n,\mathrm{MPC}}^{s})(2n+1)P_{n}(\mu )|d\mu \;=\int |(g_{3,\mathrm{MHG}}^{s}-g_{3,\mathrm{MPC}}^{s})\frac{7}{2}(5\mu^{3}-3\mu )+(g_{4,\mathrm{MHG}}^{s}-g_{4,\mathrm{MPC}}^{s})+\ldots |d\mu \;=\int |(P^{\mathrm{MHG}}(g_{1}^{s},g_{2}^{s},\mu )-P^{\mathrm{MPC}}(g_{1}^{s},g_{2}^{s},\mu )|d\mu .$$ (9)

$W_{s}$ is maximal for a single scattering event and decreases to 0 as the number of scattering events increases because both phase functions tend toward isotropy. For a more forward peaked phase function, represented by high $g_{1}$ and $g_{2}$ values, $W_{s}$ will decrease more slowly as a function of the number of scattering events than for a more isotropic phase function represented by low $g_{1}$ and $g_{2}$ values. The weighing function $W_{s}$ can thus well capture the two characteristics mentioned above.

**Fig. 6:**
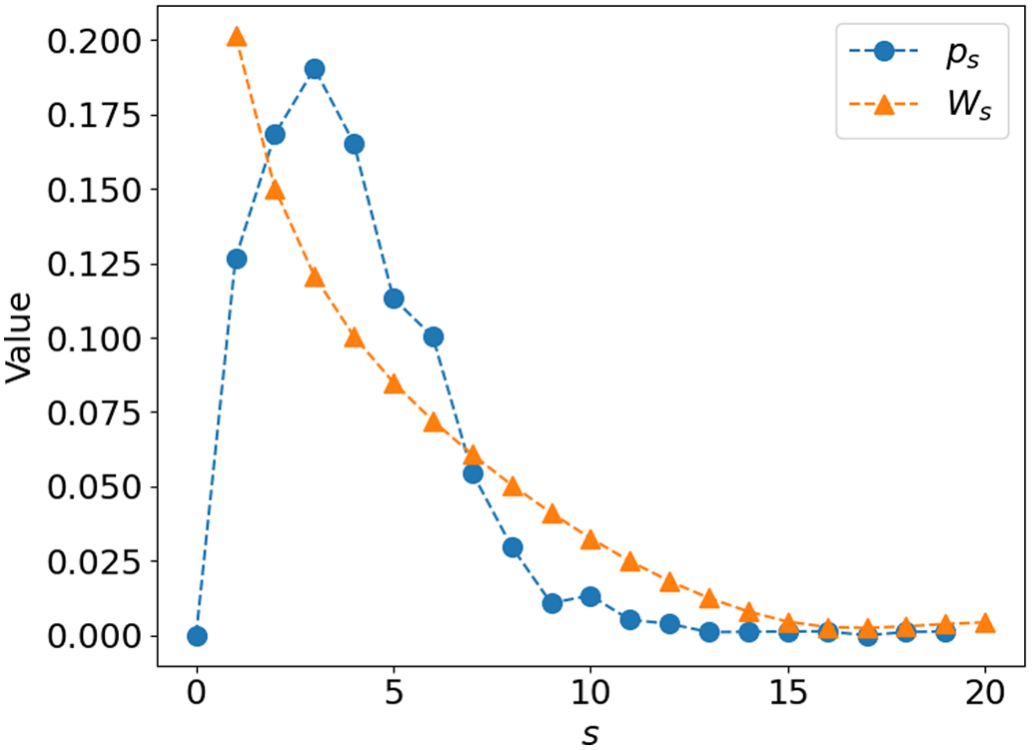
Probability density function of the number of scattering events of the collected photons at an SDS of 0.4 mm and a tilt angle of 40 deg and weight of the higher-order moments as a function of the number of scattering events.

Certainly, scattering phase functions other than the MHG and MPC could have been used to calculate $W_{s}$. However, such scattering phase functions, which can independently adjust $g_{1}$ and $g_{2}$ within a certain range while being constrained by the fact that they must satisfy the positivity constraint (p>0), are not trivial to generate. After defining this parameter, the MC simulation program was modified to calculate the distribution of s-scattered photons ($p_{s}$) at the level of each detection fiber. A typical distribution of s-scattered photons ($p_{s}$) for an SDS of 0.4 mm and a tilt angle of 40 deg for the IOPs of case #1 is shown in Fig. 6 (blue curve) with its corresponding $W_{s}$ (orange curve). Then, the proposed criterion (F) to evaluate the contribution of these higher-order moments to the diffuse reflectance is given by

$$F=\sum_{s=1}^{N}p_{s}W_{s},$$ (10)

which corresponds to the sum of the distribution of the s-scattered photons weighed by $W_{s}$, where N is the maximum number of scattering events experienced by the detected photons. The F value represents the relative contribution of the higher-order moments to the backscattered signal at a detection fiber. A small F value indicates that the photons have scattered enough times that the contribution of the higher-order moments to the reflectance is marginal. Conversely, an F value close to 1 indicates that the contribution of the higher-order moments is very high. This means that, by taking into account the forward-peaked aspect of the initial phase function, the SDS is too small to allow the detected photons to have undergone enough scattering events to make the effective phase function isotropic enough. In such a case, higher-order similarity relations must be verified to adequately model the scattering.

**Fig. 7:**
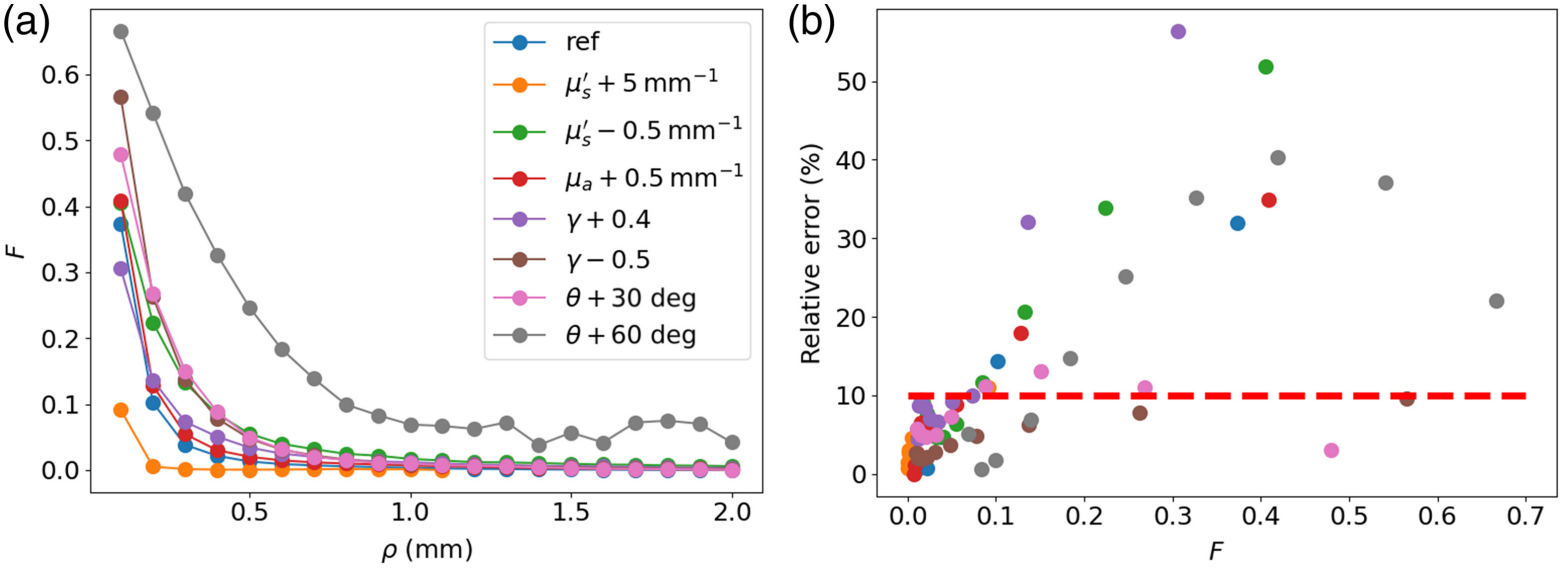
(a) F value as a function of SDS and (b) relative error as a function of criterion value for a wide range of IOP values and optical setup geometries. Baseline values are $\mu_{s}^{\prime }=0.2\;{\mathrm{mm}}^{-1}$, γ=1.5, and $\mu_{a}=0.2\;{\mathrm{mm}}^{-1}$.

To determine the value below which F must remain for the second-order approximation to be valid, a wide variety of IOPs and optical setup geometries were tested. Figure 7(a) shows the F value as a function of SDS for different IOP combinations and different tilt angles. Figure 7(b) shows the F values as a function of the relative error ($|R_{\mathrm{MHG}}-R_{\mathrm{MPC}}|/R_{\mathrm{MHG}}$) due to higher-order moments when the reflectance is simulated with the MPC and MHG phase functions with identical ($g_{1},g_{2}$) moments. The reference case is for the properties of case #1 with a tilt angle of 0 deg. Although only eight examples are given here, many more were tested, and the results presented here are representative of the general trends observed.

We see that the F value increases as $\mu_{s}^{\prime }$ decreases and as $\mu_{a}$ increases. This is because a lower $\mu_{s}^{\prime }$ value corresponds to fewer scattering events of the detected photons, which increases the contribution of the higher-order moments. On the other hand, higher $\mu_{a}$ values correspond to shorter path lengths, so the collected photons will have scattered fewer times. The effect of increasing the tilt angle also increases the F value, meaning that larger SDSs are required to achieve a similar F value. The subdiffusive parameter γ is the only parameter that does not seem to have a direct relation with F. Figure 7(b) shows that the relative error in the reflectance due to the higher-order moments remains below 10% when the F value is below 10%. The criterion proposed by Bevilacqua and Depeursinge^7^ for perpendicular fibers ($\rho \mu_{s}^{\prime }>0.5$) was established using this 10% threshold on the relative reflectance error. Thus, this same threshold is used for the proposed F criterion. This implies that, for the second-order approximations to hold, the criterion F>10% must be satisfied. The purpose of the criterion is to define the minimum SDS for a given optical geometry and tissue IOPs.

## 4 Determining the Maximum SDS: Robustness Versus Sampled Volume

**Fig. 8:**
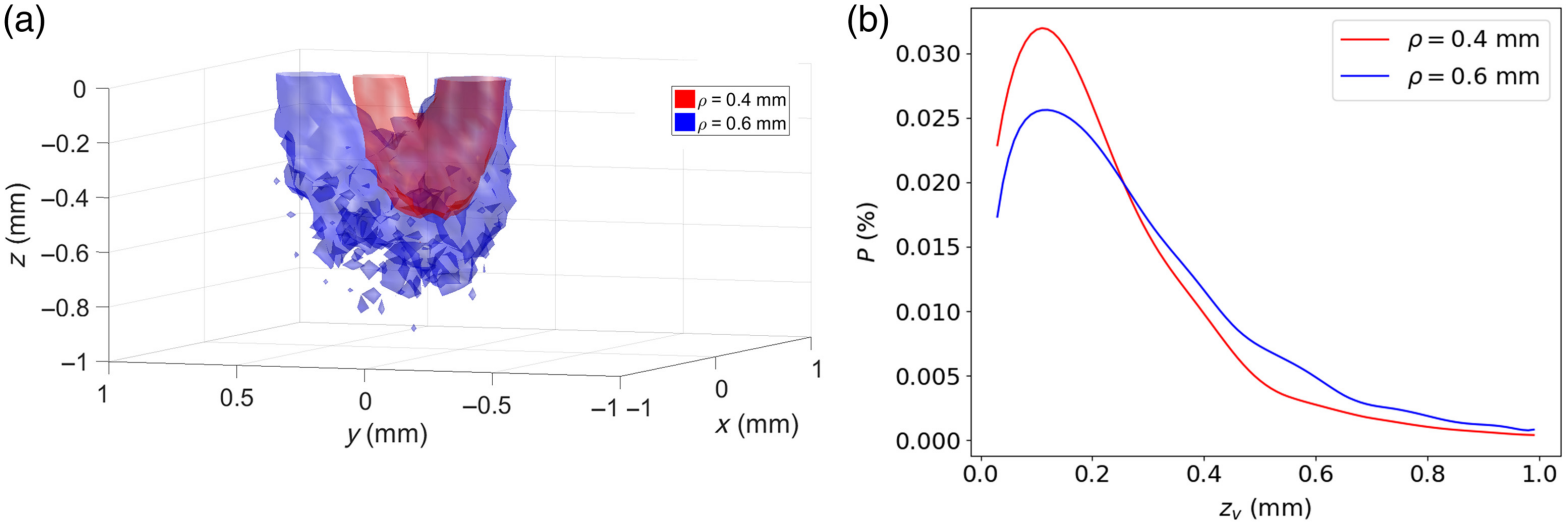
(a) Surfaces containing 70% of the scattering events of the collected photons for fibers at SDS of 0.4 and 0.6 mm and (b) distribution of the scattering events along the z axis.

Once the minimum SDS is identified for the probe geometry under consideration, the position of the other detection fibers can be determined based on the desired robustness of the inverse problem and the sampled depth. The sampling depth is estimated by recording the distribution of the scattering event positions along the z axis of the photons collected by the detection fibers. Figure 8(a) shows the 3D surface containing 70% of the scattering events for the detection fibers at SDS of 0.4 mm (in red) and 0.6 mm (in blue) for IOPs typical of biological tissue ($\mu_{s}^{\prime }=2\;{\mathrm{mm}}^{-1}$, γ=1.5, and $\mu_{a}=0.2\;{\mathrm{mm}}^{-1}$), using the MHG phase function. We see that, as the SDS increases, so does the sampling depth within the tissue. By summing over the (x,y) plane, the distribution of the scattering events as a function of depth is represented in Fig. 8(b). To represent the sampled depth with a scalar value, the depth over which 80% of the scattering events take place is used.^28^^,^^29^

**Fig. 9:**
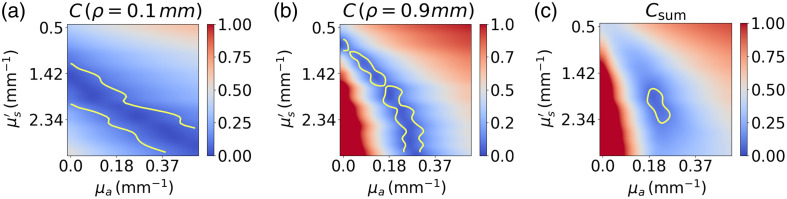
Cost function value in the $\left(\mu_{s}^{\prime },\mu_{a}\right)$ plane at SDS of (a) 0.1 mm, (b) 0.9 mm, as well as for (c) the average of 10 fibers evenly spaced between 0.1 and 2 mm. The yellow contour line delimits the region where the cost function value is smaller than 0.1.

**Fig. 10:**
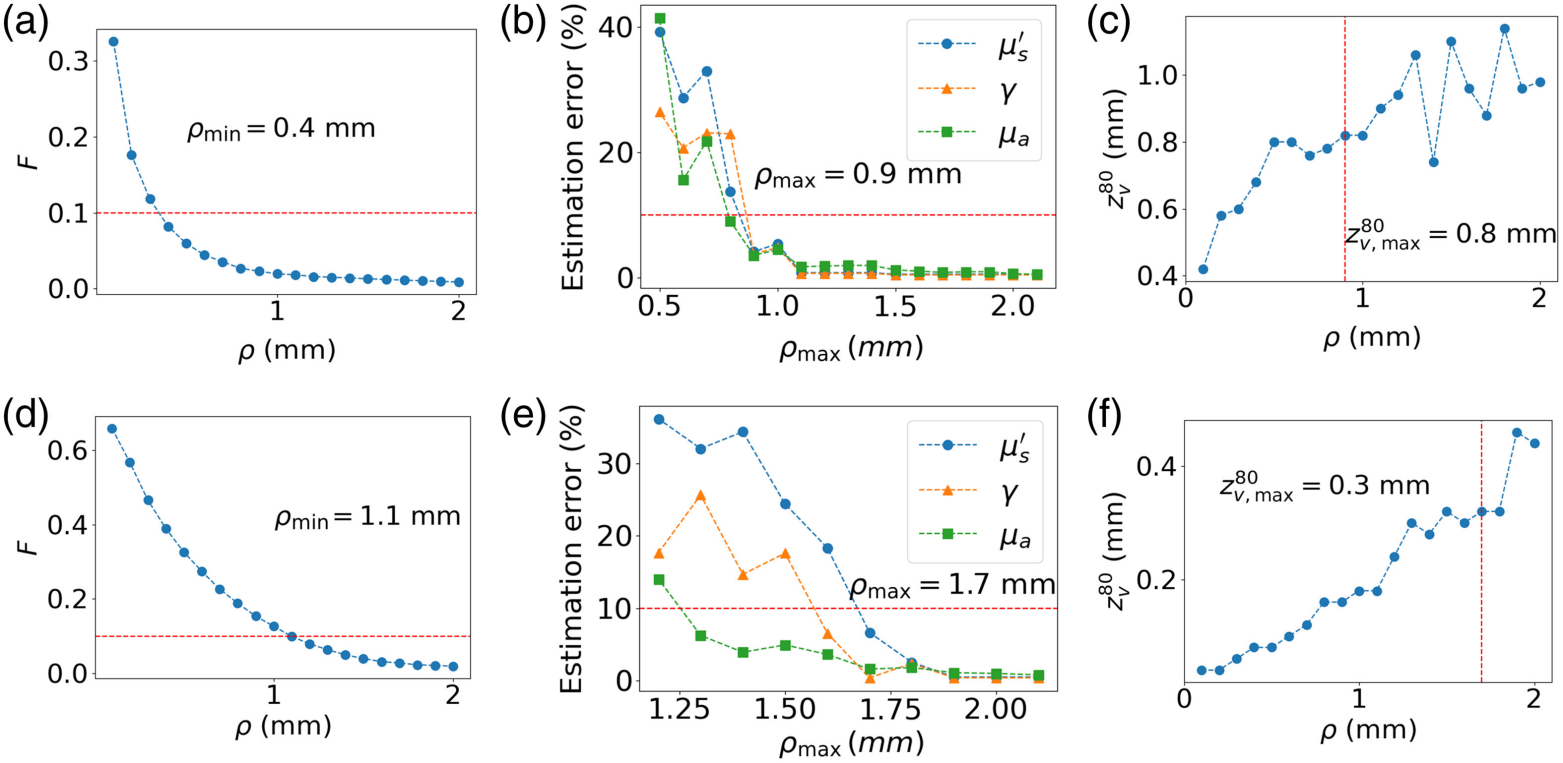
Steps for evaluating the minimal sampling depth for a considered fiber geometry. The top row considers the case of fibers perpendicular to the surface, and the bottom row fibers tilted at a 60-deg angle. (a) The computation of criterion F to identify the minimal SDS. (b) The robustness analysis as a function of SDS to identify the maximal SDS using the minimal SDS identified previously. (c) The estimation of the sampling depth at the maximal SDS.

The higher the number of measurements and the more varied the information, e.g., using different SDS or tilt angles, the more robust the estimation of the IOPs. To demonstrate this, synthetic data are generated by adding 10% white Gaussian noise to the simulated reflectance, and the IOPs are estimated for different distributions of detection fibers. Specifically, the minimum SDS is set to $\rho_{\min}=0.4\;\mathrm{mm}$, and detection fibers are added every 0.1 mm, progressively increasing the maximum SDS ($\rho_{\max}$). For each case, the process of generating synthetic data and solving the inverse problem is repeated 10 times to obtain a statistically significant result on the estimation error. Figure 10 shows that as $\rho_{\max}$ increases, the estimation error decreases. This is because, as light travels farther into the tissue, absorption has a larger impact on the DRS signal. The effect of this phenomenon on the inverse problem can be noticed by observing the cost function (C) defined in Eq. (10). Figure 9 shows the cost function in the $\left(\mu_{s}^{\prime },\mu_{a}\right)$ plane. We see that, for a single detection fiber, there is a range of possible solutions that minimize the cost function. As the SDS increases, the area representing the minimum of the cost function (shown in dark blue) rotates in the $\left(\mu_{s}^{\prime },\mu_{a}\right)$ plane, where the LUT in both axes is meant to cover realistic coefficient values for biological tissues in the 500 to 750 nm range.^13^ As the total cost function is the average of all detection fibers, the shape of the cost function becomes more convex using both fibers and better defined than when a single detection fiber is considered. Because the experimental data contain noise, the solution identified by the inverse solver is susceptible to moving around in the minimal region of the cost function, represented by the yellow contour lines. Using the information from many fiber combinations, this minimal region becomes more convex and smaller, which results in a better robustness of the inverse solver. More details about the influence of probe geometry on the shape of the cost function are given in part 2 of this paper. Furthermore, it is worth mentioning that the exact shape of the cost function for each fiber combination depends on the values of the solution. For example, in the case of a higher absorption, the minimum region of the cost function will narrow faster as a function of SDS because of the greater impact of absorption on the reflectance values.

The position of the maximum SDS thus depends on both the desired robustness of the inverse problem as well as on the acceptable compromise for the sampled depth. This is because greater robustness requires the use of fibers with larger SDS, which increases the volume sampled by light within the tissue. In these experiments, where 10% white Gaussian noise is added to the synthetic data, a 10% estimation error in IOP determination is considered acceptable for the choice of $\rho_{\max}$.

## 5 Influence of IOPs on Probe Design

One problem that must be emphasized when determining the minimum and maximum SDS for a new DRS probe design to neglect the contribution of the higher-order moments and obtain a robust calculation of the IOPs of the medium is that this determination depends on the IOPs themselves. In the context of biomedical applications, the IOPs are generally not known prior to the DRS acquisition. One solution to this problem is to consider a limit case, i.e., realistic IOP values that would require the largest minimum SDS. As seen in Fig. 7(a), this represents a tissue with a high $\mu_{a}$ and a low $\mu_{s}^{\prime }$ value. Based on a literature review of IOPs in biological tissues,^13^ a reasonable limiting case is $\mu_{s}^{\prime }=1\;{\mathrm{mm}}^{-1}$, $\mu_{a}=0.2\;{\mathrm{mm}}^{-1}$, and γ=1.9, labeled case #2.

To determine the minimum sampled depth of the probe as a function of the fiber tilt angle for case #2, the three steps are carried out, namely: (1) identify $\rho_{\min}$ based on the criterion F>0.1, (2) identify $\rho_{\max}$ that guarantees an estimation error linear with the level of added noise, and (3) evaluate the sampled depth at $\rho_{\max}$. Figure 10 shows the results of these three steps using tilt angles of 0 and 60 deg.

We see that, for perpendicular fibers, $\rho_{\min}=0.4\;\mathrm{mm}$ and $\rho_{\max}=0.9\;\mathrm{mm}$, which correspond to a sampling depth of 0.8 mm. By increasing the fiber tilt angle, the minimum SDS is increased to 1.1 mm because the photons undergo fewer collisions for the same SDS. The $\rho_{\max}$ value is 0.6 mm farther away, which is a similar result to the one obtained for the case at 0 deg. Although the SDSs are larger for the tilted-fiber geometry, the sampled depth is reduced to 0.3 mm, which is an improvement of almost three times. This highlights the compromise that must be made between minimizing the sampling depth and the sampling surface. If a tissue is known to be homogeneous in the (x,y) plane and inhomogeneous in the z axis, which is typical for the multilayered organization of epithelial tissue, then it is preferable to use tilted fibers to minimize the sampling depth as much as possible. By minimizing the sampling depth through the use of tilted fibers, it may be possible to limit the volume of the collected photons within the superficial layer of the tissue. This is of particular interest because it was shown that a false assumption of homogeneous tissue when photons interact with two layers can drastically affect the validity of the IOP estimation.^16^

### 6 Conclusions

In this work, a framework for the design of tilted-fiber geometry srDRS probes that can quantitatively estimate the IOPs of living tissue is presented. The DRS literature rarely distinguishes between a qualitative and a quantitative assessment of the IOPs. For the former, there are no constraints on the optical setup’s geometry, and the previously described considerations in the phase function modeling are of little importance because the goal is simply to observe relative changes in the reflectance signal, which can be related to changes in tissue optical properties. These relative changes can be observed over time, for example, in the case of bedside monitoring, or among different tissue states for diagnostic applications. On the other hand, quantitative estimation of the tissue IOPs has the advantage of providing reliable information about the biochemistry and histoarchitecture of tissues, as well as allowing comparison across different experimental setups. Quantitative estimation imposes additional constraints on the optical setup geometry, and a careful analysis of the conditions to approximate the scattering phase function with a limited number of Legendre moments is necessary.

In this work, a criterion based on the distribution of scattering events of the collected photons and the convergence of the phase function toward isotropy is proposed. This criterion guarantees the effect of the moments of order higher than 2 on the reflectance curve of less than 10%. Although the criterion is presented here in the context of the second-order approximation of the phase function, it can be easily adapted for any approximation order. The criterion is used to determine the minimum SDS as a function of probe geometry and tissue IOP. In contrast, the shortest maximum SDS can then be selected by analyzing the effect on both the accuracy of the quantitative calculation of IOP and the size of the sampled tissue volume. Specifically, the maximum SDS is defined by the acceptable compromise between IOP estimation robustness and sampling depth. The photons collected by the fiber at the largest SDS are the ones that travel the deepest in the tissue, so this fiber defines the complete probe’s sampling depth. To evaluate the sampling depth, a new metric was proposed ($z_{v}^{80}$), which represents the depth over which 80% of the collected photons’ scattering events take place.

Numerical results emphasize that increasing the tilt angle from 0 to 60 deg tends to reduce the sampling depth by a factor of ∼2.6. This comes at the expense of a larger lateral sample area (i.e., parallel to the tissue surface) as the maximum SDS increases by a factor of ∼1.8. Therefore, when designing new srDRS probes, several decisions should be made regarding the best shape of the light-sampled volume. It is worth noting that the sampling depth could be further reduced by incorporating higher similarity relations in the numerical model, such as δ and ϵ.^21^^,^^22^ These approaches seem promising but have the major inconvenience of adding more unknowns to the inverse problem, which complicates its resolution. This work provides a solid framework for designing efficient srDRS optical setups to quantitatively estimate IOPs. As reported IOP tissue values vary widely in the literature and knowing that the distinction between qualitative and quantitative IOP estimations is often not clearly stated in DRS publications, we hope that this work will help to obtain reliable quantitative estimation of tissue IOP.

## Data Availability

Data and code developed in this study are available upon reasonable request to the corresponding author.
